# FINANCIAL EXPLOITATION VULNERABILITY IN OLDER AGE: INTERPERSONAL AND BRAIN MECHANISMS

**DOI:** 10.1093/geroni/igae098.0368

**Published:** 2024-12-31

**Authors:** Duke Han

**Affiliations:** University of Southern California, Los Angeles, California, United States

## Abstract

Financial exploitation vulnerability in older age can be viewed as an intersectional model of multiple potentially contributing features that can vary from individual predisposing characteristics to interpersonal dynamic factors. Here we present recent work from our lab focused on two mechanisms. In Study 1, thickness of the entorhinal cortex as measured by a 7-Tesla brain MRI scan was associated with a measure of perceived financial vulnerability in 97 older adults without any significant cognitive impairment in models adjusted for demographics and cognition (beta=-0.05, SE=0.02, p<0.05). The entorhinal cortex is well-known for its role as the first brain region to show signs of deterioration in preclinical Alzheimer’s Disease; therefore, findings suggest early age-associated pathological brain changes may underlie financial vulnerability. In Study 2, financial vulnerability was associated with depth of relationships, but not breadth of relationships, in 115 older adults without any significant cognitive impairment after covarying for demographics, depressive/anxious symptoms, and cognition (beta=-.08, SE=.03), p<.01). Findings argue for the importance of considering interpersonal relationship dynamics when assessing financial vulnerability. Together, these studies argue for the importance of considering multiple factors and different levels of analysis when considering financial exploitation vulnerability in older age.

